# Enhanced Performance of Carbon Molecular Sieve Membranes Incorporating Zeolite Nanocrystals for Air Separation

**DOI:** 10.3390/membranes11070489

**Published:** 2021-06-29

**Authors:** Chong Yang Chuah, Kunli Goh, Tae-Hyun Bae

**Affiliations:** 1 Singapore Membrane Technology Centre, Nanyang Environment and Water Research Institute, Singapore 637141, Singapore; chongyang.chuah@ntu.edu.sg (C.Y.C.); gohkunli@ntu.edu.sg (K.G.); 2 Department of Chemical and Biomolecular Engineering (BK21 Four), Korea Advanced Institute of Science and Technology, Daejeon 34141, Korea

**Keywords:** zeolite, carbon molecular sieve membrane, air separation, Robeson upper bound, mixed-matrix

## Abstract

Three different zeolite nanocrystals (SAPO-34, PS-MFI and ETS-10) were incorporated into the polymer matrix (Matrimid^®^ 5218) as polymer precursors, with the aim of fabricating mixed-matrix carbon molecular sieve membranes (CMSMs). These membranes are investigated for their potential for air separation process. Based on our gas permeation results, incorporating porous materials is feasible to improve O_2_ permeability, owing to the creation of additional porosities in the resulting mixed-matrix CMSMs. Owing to this, the performance of the CMSM with 30 wt% PS-MFI loading is able to surpass the upper bound limit. This study demonstrates the feasibility of zeolite nanocrystals in improving O_2_/N_2_ separation performance in CMSMs.

## 1. Introduction

Air, which mainly consists of oxygen (O_2_) and nitrogen (N_2_), is an important element for various industries and chemical processes. In general, fuel combustion is typically more advantageous if oxygen-enriched air (OEA) is utilized in comparison to atmospheric air, in order to increase the overall heating value and combustion efficiency [1,2,3]. Besides, high-purity oxygen is used in the several applications, including but not limited to, sewage treatment plants, medical industries, and indoor air quality (IAQ) management in production buildings [4]. Nitrogen-enriched air, on the other hand, is utilized mainly in food preservation to extend the expiry date of oxygen-sensitive food products as well as fire extinguishers used in the coal extraction process, considering its inert properties [5,6].

Conventionally, in industrial processes, the production of high-purity (>99.5 vol%) oxygen and nitrogen from air can be achieved by cryogenic distillation or pressure swing adsorption (PSA). Although these technologies have been present for at least 70 years with a daily production volume of 100 tonnes, high capital cost and large energy penalty challenge the need to consider an alternative process for air separation [7,8]. This attracted substantial research interest towards membrane-based separation. Today, membrane-based high-purity oxygen production is limited to only 25 tonnes per day [9,10], but its practical feasibility due to its simplicity in its operation, cost effectiveness and small plant footprint are competitive advantages over cryogenic distillation and PSA.

To date, membrane materials for the gas separation process are mainly dominated by polymeric materials due to their well-established large-scale synthesis together with the capability of adopting different configurations (spiral wound or hollow fiber). Nevertheless, polymeric membranes are well known for their trade-off limitation between permeability and selectivity as described by Robeson [11,12], given that gas transport in dense polymeric membranes follows the solution-diffusion mechanism [13]. Thus, current research efforts are mostly focused on overcoming this trade-off limitation. Interestingly, the development of carbonized polymeric precursors is a feasible approach in circumventing this limitation [14,15,16,17], through the creation of carbon molecular sieve membranes (CMSMs). In general, pyrolysis of polymer precursors under various treatment conditions gives large graphitic domains, which have been showcased to drastically improve gas permeability without sacrificing the mixed-gas selectivity considerably [18,19,20]. Nevertheless, the performance of CMSMs is heavily influenced by the selection of polymer matrix. For instance, CMSMs derived from highly permeable polymers demonstrate extraordinarily high gas separation performances, but the synthesis of these polymers is typically painstaking and laborious to ensure sufficiently large molecular weight for mechanical stability in the subsequent carbonization process [21,22,23].

Hence, in this work, we leverage a different approach, which is to tune the air separation performance by adopting mixed-matrix strategy into the fabrication of CMSMs. This approach involves the incorporation of zeolite nanocrystals as filler materials into the polymeric membrane, using Matrimid^®^ 5218 polyimide as the precursor. Matrimid^®^ 5218 is selected due to its commercial availability in comparison to PIM-1 and 6FDA-diamine polymers that possess high intrinsic gas permeability, which require extensive monomer purification [24]. Besides, Matrimid^®^ has a high O_2_/N_2_ selectivity (c.a. 5.9) [25], such that a high performance can be realized simply by improving permeability without sacrificing the O_2_/N_2_ selectivity, which can be done by incorporating highly porous filler materials. In addition, research on mixed-matrix CMSMs for air separation process is scarce as compared to CO_2_-based separation process [15,21,26]. Herein, we chose three different types of zeolites nanocrystals having different pore sizes, namely Engelhardt Titanosilicate (ETS-10), silicalite-1 (pure silica MFI, PS-MFI) and silicoaluminophosphate (SAPO-34), as our filler materials. Based on our gas permeation results, PS-MFI at 30 wt% loading in carbon matrix of Matrimid^®^ 5218 membrane was able to surpass the upper bound limit for O_2_/N_2_ separation.

## 2. Materials and Methods

### 2.1. Materials

Matrimid^®^ 5218 polymer was obtained from Huntsman Corporation (Conroe, TX, USA). Titanium dioxide (TiO_2_, Aeroxide P25) was obtained from Jebsen and Jessen Ingredient (S) Pte Ltd. (Singapore). Aluminum isopropoxide (Al(i-C_3_H_7_O)_3_), phosphoric acid (85 wt% aqueous solution, H_3_PO_4_), sodium hydroxide (NaOH), tetraethyl orthosilicate (TEOS), tetraethylammonium hydroxide (35 wt% aqueous solution, TEAOH), colloidal silica (LUDOX TM-40, 40 wt% suspension in H_2_O, SiO_2_) were purchased from Sigma Aldrich (St. Louis, MO, USA). Tetrapropylammonium hydroxide (aqueous solution with 40 wt% TPAOH) was obtained from Alfa Aesar (Tewksbury, MA, USA). Chloroform and potassium fluoride (KF) were brought from VWR (Philadelphia, PA, USA). Distilled water, H_2_O was synthesized in-house.

### 2.2. Synthesis Procedure for SAPO-34, PS-MFI and ETS-10

SAPO-34 [27]: SAPO-34 was synthesized using a dry-gel conversion method. In this synthesis, TEOS and Al(i-C_3_H_7_O)_3_ were used as the aluminum and silicon source, respectively. In subsequent steps, Al(i-C_3_H_7_O)_3_, H_3_PO_4_, TEOS and TEAOH were added sequentially into H_2_O to give a solution mixture. During each process, the solution was allowed sufficient agitation (3 h) to ensure that the resulting gel remained homogeneous. The molar composition of the prepared gel was calculated as 1.00 Al_2_O_3_/0.30 SiO_2_/1.00 P_2_O_5_/1.00 TEAOH/52.00 H_2_O. The solvents (H_2_O and ethanol) were removed by heating at 80 °C, so as to form a dry gel. After the gel was sufficiently dried, H_2_O was added into the solution in order to achieve the desired ratio of 3:1 (H_2_O/Al_2_O_3_), which was then followed by a hydrothermal synthesis at non-agitated conditions (220 °C for 24 h). Centrifugation was used the remove any potential presence of any undesirable residual reactants that could be present in SAPO-34 crystals. This process was reported for at least three times with the use of distilled water, prior to drying at 110 °C overnight. The residual template (TEAOH) that was present in the dried SAPO-34 samples was removed via a calcination process in air at 500 °C for 6 h.

PS-MFI [28]: Pure silica MFI was developed as described below. TEOS was used as the silica source, as similar to SAPO-34. First, TEOS was added into a solution (that contained TEAOH) while maintaining an agitation process. The solution turned completely transparent after an additional 1 h of stirring. The solution was then allowed to agitate further for 24 h after H_2_O was added into the solution. The molar composition of the synthesized gel was determined as 1.00 TEOS/0.36 TPAOH/20.00 H_2_O. In the following step, PS-MFI crystals were obtained via a continuous agitation at 95 °C under a duration of 48 h. The precipitated crystals were washed with copious amount of H_2_O via a repetitive centrifugation process. The residual template (TPAOH) that was potentially present in the crystals was calcined in air at 550 °C for 8 h.

ETS-10 [29]: ETS-10 was synthesized based on the procedure as provided. Colloidal silica as well as TiO_2_ were used as the source of silicon and titanium, respectively. First, a suspension was created by dispersing TiO_2_ into H_2_O. The solution was agitated for 1 h after the addition of NaOH and KF, followed by an additional 6 h of stirring after colloidal silica was added into the mixture. At this stage, the molar composition of the solution was identified as 1.00 SiO_2_/0.30 Na_2_O/0.20 TiO_2_/0.15 KF/16.30 H_2_O. Subsequently, hydrothermal reaction was conducted at 215 °C for 72 h under static condition. The ETS-10 crystals were collected via a centrifugation process for at least three times using distilled water, before drying the samples at 60 °C overnight.

### 2.3. Development of Mixed-Matrix Carbon Molecular Sieve Membranes (CMSMs)

Polymeric precursor membranes were created in a flat sheet configuration using a solution-casting approach. As an illustration, to fabricate a polymeric membrane without the addition of zeolite nanocrystals, dissolution of Matrimid^®^ 5218 into a chloroform solution was made to prepare a dope solution. For the case of mixed-matrix membranes (MMM), the dope solution was developed by first dispersing the zeolite fillers into the solution containing chloroform. Sonication horn (Qsonica, Q125, Newtown, CT, USA) was used to potentially minimize nanocrystals’ aggregation. Next, Matrimid^®^ 5218 powder was added into the suspension. At least 24 h was set to stir the dope solution before casting the membranes. Using a casting knife, the membranes were casted onto glass plate. The glass plates were placed in a glove bag, where the environment was filled with chloroform vapor during the fabrication process to control solvent evaporation. The membranes were eventually placed in vacuum oven at 160 °C overnight. Horizontal tube furnace by Carbolite GERO (CTF 12/10/900) was used to carbonize the polymeric membranes to afford CMSMs. In this study, Argon (99.995%) that was purchased from Airliqude Singapore was utilized. The system was purged for the minimum duration of 1 h to displace any left-over oxygen that was present in the system. The carbonization process was conducted under the following profile: (1) 2 °C min^−1^ ramp (25 °C to 380 °C) for 0.5 h; (2) 0.5 °C min^−1^ ramp (380 °C to 550 °C) for 2 h. The CMSMs were cooled to ambient condition prior to sample retrieval. 

### 2.4. Characterizations

NOVATouch LX2, a volumetric gas sorption analyzer, was used to assess the porosity properties of SAPO-34, PS-MFI and ETS-10 (Quantachrome, Boynton Beach, FL, USA). In a typical case, outgassing the zeolite samples was conducted at 250 °C for 24 h under high vacuum prior to the investigation of physisorption isotherm at 77 K (−169 °C), with the use of nitrogen (N_2_) as the adsorbate. On the other hand, pure component adsorption (O_2_ and N_2_, respectively) of SAPO-34, PS-MFI and ETS-10 was conducted at 35 °C, where the temperature was controlled using a water circulator. The samples were activated at the same condition as described above. The isosteric heat of adsorption (-*Q*_st_) of O_2_ and N_2_ of these porous materials was computed with the data from the isotherms measured at two temperatures (25 and 35 °C). This was calculated using the Clausius–Clapeyron equation, as described by Equation (1). *q*, *T* and *P* were described as adsorbed quantity (mmol g^−1^), absolute temperature (Kelvin) and pressure (bar), respectively. As the effect of temperature *-Q*_st_ could be neglected in most circumstances [30], a well-defined mathematical solution could be obtained when Equation (2) (single-site Langmuir) was used. Fitting parameters for the adsorption isotherm at 25 and 35 °C [31,32] can be found in the Supplementary Information (Tables S1 and S2). *b*, *P* and *q*_sat_ were described as Langmuir constant (bar^−1^), pressure (bar) and saturation loading (mmol g^−1^), respectively.

(1) $$Q_{st}=RT^{2}\left(\frac{\partial lnP}{\partial T}\right)$$

(2) $$q=\frac{q_{sat}bP}{1+bP}$$

The pore size distribution of CMSM and mixed-matrix CMSMs were analyzed with the use of CO_2_ adsorption at 0 °C, measured by the volumetric gas sorption analyzer as mentioned in Section 2.4. Similarly, the carbonized membranes were degassed at 250 °C for 24 h before the measurement. Subsequently, the CO_2_ adsorption isotherm was modelled with a Dubinin–Radushkevich (DR) equation, as illustrated in Equation (3). In this expression, *V*, *V*_o_, *E*_o_ and *β* were the amount of CO_2_ adsorbed (at specified relative pressure (*P/P_o_*) and temperature (*T*)), the micropore volume, characteristic adsorption energy and affinity coefficient (0.35 for CO_2_), respectively [33]. This process allowed the micropore volume, micropore size and micropore surface area to be computed. Such measurements were conducted as N_2_ physisorption at 77 K was unable to be determined in CMSMs as negligible N_2_ adsorption was typically observed [16]. This behavior potentially indicated the formation of ultramicropores during the membrane carbonization process, as reported elsewhere [34].

(3) $$V=V_{o}exp\left[-k{\left(\frac{RT}{E_{o}\beta }\right)}^{2}{\left(\ln\frac{P_{o}}{P}\right)}^{2}\right]$$

The verification of the crystallinity of the zeolite nanocrystals was performed with the use of powdered X-ray diffraction (PXRD). This measurement was conducted with the use of Bruker D2 phaser, which contained a diffractor with a CuKα radiation (1.5418 Å) (Billerica, MA, USA). Such analysis was conducted at an ambient condition. The measurement was conducted at 2*θ* range from 5° to 40°, using step size of 0.02°. Field-emission scanning electron microscope (FESEM) was performed to observe the particle morphologies. The acceleration voltage (Joel, JSM6701, Tokyo, Japan) was set as 5 kV. Image analysis tool (Nano Measure) was used to determine the particle size distribution of the zeolite nanocrystals, based on the FESEM images. The thermal stabilities of the zeolite nanocrystals were conducted using thermogravimetric/differential thermal analyzer (TG/DTA, TA Instrument, SDT Q600, New Castle, DE, USA). The ramping profile was set at 10 °C min^−1^ from 40 to 800 °C, with the use of nitrogen gas set at 0.1 L min^−1^.

### 2.5. O_2_ and N_2_ Permeation Analysis

Constant pressure-variable volume system by GTR Corporation was utilized to perform the O_2_ and N_2_ permeation test. Air (Purity of O_2_ and N_2_ of 99.8% and 99.995% at 21/79 vol ratio, Airliquide, Singapore) mixture and helium (99.995%, Airliquide, Singapore) were utilized in this setup for the measurement. First, the membrane was mounted onto the permeation cell, with the temperature of the measurement maintained at 35 °C. Membranes are subjected to the flow of air (test gas, at upstream) and helium (at downstream) in a continuous manner. Flow rates were controlled using mass flow controllers. The permeated gases were swept by helium gas into a gas chromatograph at a set time interval with the aid of a gas sampler. Once the amount of gas permeated did not demonstrate large fluctuation, the permeability and selectivity of the studied gases were calculated. At least three different samples were measured to confirm the reproducibility of the permeation results.

### 2.6. O_2_ and N_2_ Solubility-Diffusivity Analysis

The gas sorption analyzer as reported earlier in Section 2.4 was adopted to determine the gas adsorption of both O_2_ and N_2_, with the same activation condition. At the desired pressures (O_2_: 0.21 bar; N_2_: 0.79 bar), the solubility was calculated by Equation (4). The amount of gas adsorbed normalized by the mass of CMSMs, desired pressure as well as carbon membrane’s density were expressed as *q*, *P* and *ρ*, respectively. Analytical balance (Mettler Toledo, ME204) that was integrated with the accessories (density kit) was performed to obtain the density of the CMSMs. The diffusivity of the gas in CMSMs was then determined by dividing permeability with solubility.

(4) $$S=\frac{q\rho }{P}$$

### 2.7. Filler Enhancement Index, F_index_

The effectiveness of a filler in mixed-matrix CMSMs can be calculated with the use of Filler Enhancement Index (*F_index_*) [10]. This equation was described with the use of Equation (5). *P*_composite_ and *P*_unfilled_ referred to O_2_ permeability while α_composite_ and α_unfilled_ referred to O_2_/N_2_ selectivity of mixed-matrix and pure polymeric CMSMs, respectively. *η* referred to the enhancement coefficient, which was obtained from the gradient of the O_2_/N_2_ upper bound defined in 2008 as described by Robeson, which is determined as 5.666 [12]. In this calculation, utilization of different zeolite nanocrystals in mixed-matrix CMSMs was computed to comprehend the effect by the zeolite fillers.

(5) $$F_{index}=\ln\left(\frac{P_{composite}}{P_{unfilled}}\right)+n\ln\left(\frac{\alpha_{composite}}{\alpha_{unfilled}}\right)$$

## 3. Results

### 3.1. Properties of Zeolite Nanocrystals

**Figure 1:**
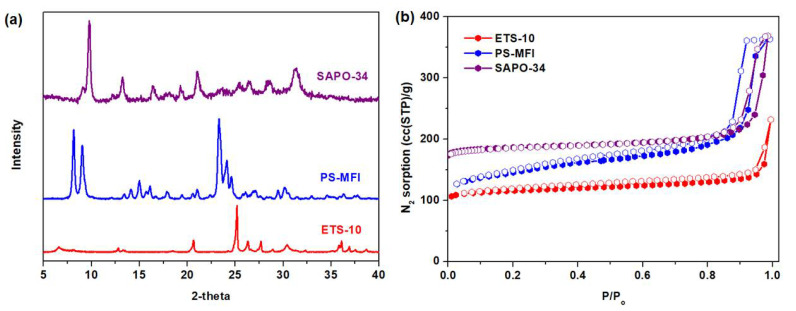
(**a**) PXRD and (**b**) N_2_ physisorption (77 K) for ETS-10, PS-MFI and SAPO-34.

**Figure 2:**
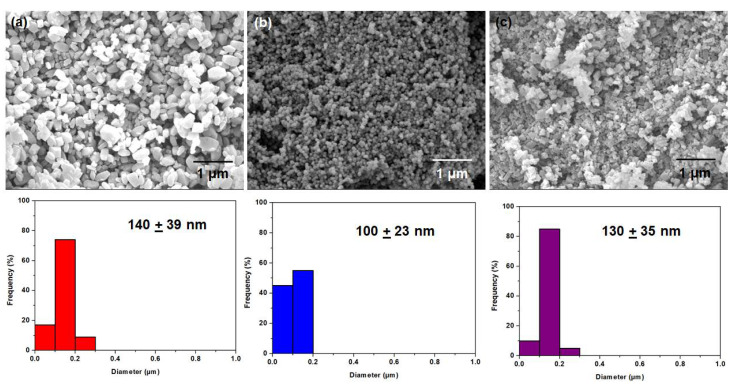
FESEM images of (**a**–**c**) ETS-10; PS-MFI and SAPO-34. Distribution of particle size was illustrated with the use of histogram.

The crystallinity of zeolite nanocrystals that were developed in this work was verified via XRD diffraction (Figure 1a). The diffraction peaks are comparable with the literature data [35,36,37], indicating the successful synthesis of SAPO-34, PS-MFI and ETS-10. Subsequently, N_2_ physisorption at 77 K (Figure 1b) depicts a typical Type 1 isotherm across the studied zeolite nanocrystals. This is attributed to its large N_2_ sorption at low *P/P_o_*, indicating large micropore volumes (Table 1) [38]. Moreover, at *P/P_o_* > 0.9, the presence of unrestricted monolayer-multilayer adsorption can be seen based on the significant increase in N_2_ sorption at this region. Such behavior is often correlated to the plausible nanocrystal creation. This deduction is further corroborated by using visual inspection from FESEM. As illustrated in Figure 2, zeolites in nanocrystal forms were indeed successfully created. Essentially, it is recommended to utilize filler particles in nanocrystals forms in the formation of mixed-matrix membranes in order to increase the interfacial areas between the fillers and polymers. The particle size of our zeolites is estimated to be ranging between 100 and 200 nm.

**Figure 3:**
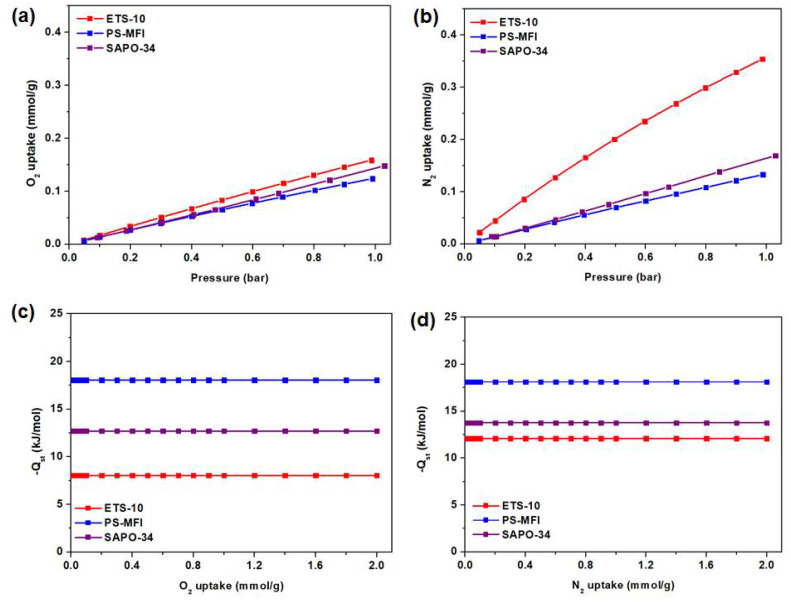
(**a**,**b**) O_2_ and N_2_ adsorption at 35 °C; (**c**,**d**) isosteric heat of adsorption (-*Q*_st_) of O_2_ and N_2_ for zeolite nanocrystals.

Subsequently, pure component gas adsorption isotherms of the zeolite nanocrystals were obtained under the pressure range from 0 to 1 bar (Figure 3a,b). The isotherm was performed at 35 °C. The -*Q_st_* calculations, on the other hand, which are typically calculated based on the measurement of at least two distinct temperatures, are calculated and summarized in Figure 3c,d. O_2_ and N_2_ isotherms at 25 °C are included in Figure S1. In general, linear gas adsorption isotherms were observed in both O_2_ and N_2_ isotherms, which suggest weak binding energy between adsorbate and adsorbent. This phenomenon is illustrated further from the analysis of *-Q_st_*, where active sites for O_2_ and N_2_ adsorption are considered homogeneous. Such behavior is not commonly observed for the case of CO_2_ adsorption where two distinctive steps (from higher to lower binding energy) can be seen [39,40]. Further comparison of the adsorption isotherms indicates that all studied zeolite nanocrystals demonstrate higher N_2_ adsorption as compared to O_2_, considering that the former possesses higher polarizability (17.6 × 10^−25^ cm^3^ vs. 15.4 × 10^−25^ cm^3^) [41]. Thus, we do not foresee these zeolites to achieve high O_2_/N_2_ sorption selectivity. Nevertheless, as compared to recent metal-organic frameworks (MOFs), which have been reported to achieve reasonable O_2_/N_2_ separation performance, these zeolites show stronger capacities for CMSMs fabrication given their better thermal stability and reversible O_2_ adsorption [42,43,44,45].

### 3.2. Properties of Carbonized Membranes

**Figure 4:**
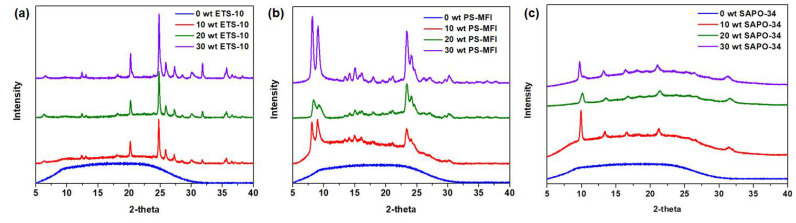
X-ray diffraction (XRD) pattern of carbonized membrane. (**a**–**c**) ETS-10; PS-MFI and SAPO-34 were added into the polymer matrix at 10, 20 and 30 wt% loading. Matrimid^®^ 5218 is used as the polymer precursor in this work.

With the successful preparation of zeolite nanocrystals, mixed-matrix membrane precursors were developed next. First, thermal stabilities of zeolite nanocrystals were determined to verify that the carbonization temperature set in this work (550 °C) is appropriate. Based on the profile in Figure S2, a substantial weight loss was not observed across all samples. The decrease in weight at initial temperatures is attributed to the evaporation of residual solvents and/or water that are present in zeolite nanocrystals. Additionally, verification of the zeolites’ crystallinity after the carbonization process was also conducted using XRD on the mixed-matrix CMSMs, as shown in Figure 4. It can be seen that the characteristic peaks of zeolite nanocrystals (with reference to Figure 1a) remain, suggesting intact crystallinity and structural integrity of the zeolite nanocrystals.

**Figure 5:**
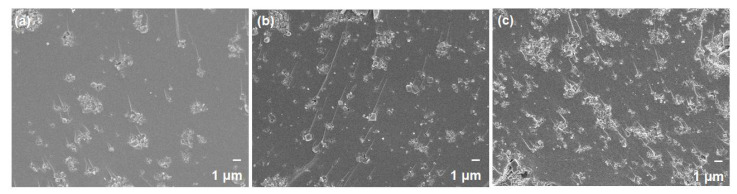

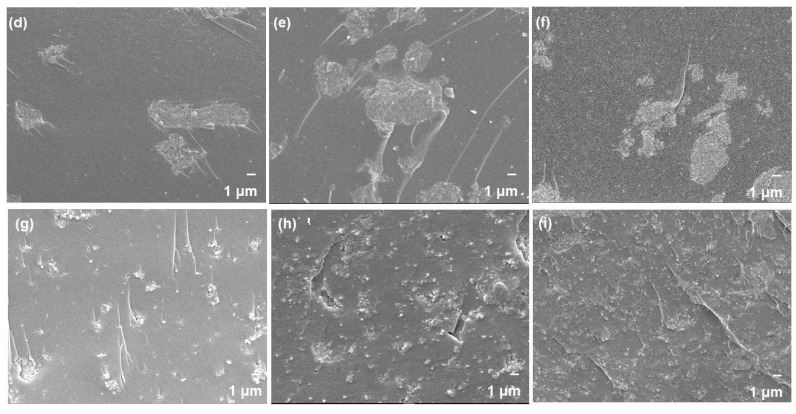
FESEM images of mixed-matrix CMSMs for (**a**–**c**) ETS-10; (**d**–**f**) PS-MFI; (**g**–**i**) SAPO-34 at 10, 20 and 30 wt%, respectively, in carbonized Matrimid^®^ 5218 membrane.

Cross-sectional morphologies of carbonized membrane with the addition of zeolite nanocrystals were imaged with the use of FESEM (Figure 5). It has been well-known that the addition of zeolites as porous fillers in mixed-matrix membranes often result in a sieve-in-a-cage morphology [46,47], considering its poor compatibility between the inorganic zeolites and polymer matrix. Nevertheless, defective voids are not visibly seen after membranes are carbonized. It is possibly due to the polymer chains undergoing thermal rearrangement during carbonization, which made it feasible to heal the interfacial defects that could be present in the mixed-matrix polymeric precursor membranes [14,15].

### 3.3. Gas Permeation Analysis of Carbonized Membranes

Measurements at 35 °C and 1 bar air feed were conducted to investigate the separation performance of the CMSMs. The results are summarized in Table 2. O_2_ permeability and O_2_/N_2_ selectivity of carbonized Matrimid^®^ 5218 membrane is reported to be 5 barrer and 5.50, respectively, which correspond to a higher O_2_ permeability with a marginal dip in O_2_/N_2_ selectivity with reference to the dense Matrimid^®^ 5218 polymeric precursor membrane [13]. The performance of carbonized Matrimid^®^ 5218 membrane is comparable with the literature results [48]. Subsequently, gas permeation analyses with systematic loadings of zeolite nanocrystals (SAPO-34, PS-MFI and ETS-10) in carbonized membrane were conducted. Based on the gas permeation result, addition of zeolite nanocrystals demonstrated substantial enhancement in O_2_ permeability by 2720%, 9200% and 3420%, respectively, at 30 wt% loading, which is possibly attributed to the substantial increase in the micropore surface area and micropore volume, as described in Table S3 (the DR plots and CO_2_ adsorption at 0 °C are provided in Figure S3). For example, incorporation of 30 wt% PS-MFI in CMSMs could increase the micropore volume and micropore surface area by 26% and 24%, respectively (Table S3). Such a phenomenon is attributed to the pore size of the studied porous fillers (ETS-10: 6–8 Å; PS-MFI: 5 Å; SAPO-34: 3.8 Å) [49,50,51,52], which allows rapid transport of O_2_ (3.46 Å) [41] and N_2_ (3.64 Å) [41] molecules through the carbon matrix of the CMSMs. Besides, based on the O_2_/N_2_ selectivity of mixed-matrix CMSM, incorporation of ETS-10 suffers the highest dip in O_2_/N_2_ selectivity, considering its highest N_2_ adsorption as compared to other porous fillers.

**Figure 6:**
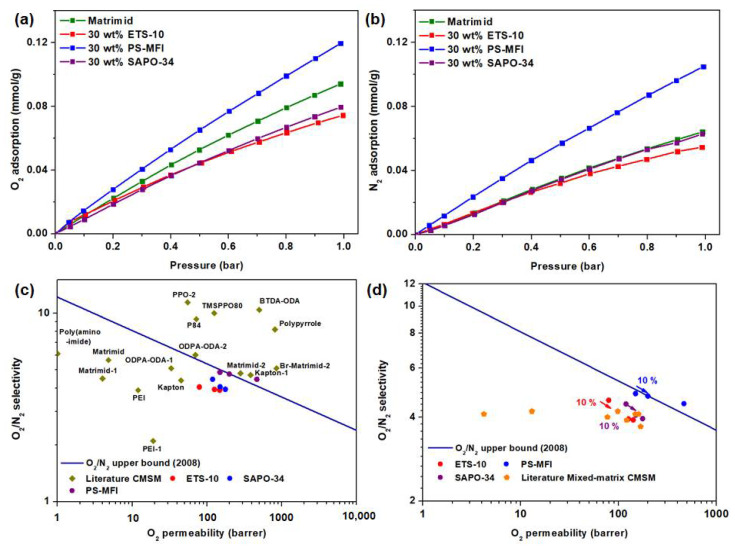
(**a**,**b**) Adsorption isotherms O_2_ and N_2_ of carbonized membrane. The measurement condition for this isotherm was set as 35 °C. (**c**,**d**) O_2_ permeabilities and O_2_/N_2_ selectivity of carbonized membrane that are reported in the literature are compared with the Robeson plot. Carbonized membrane in this work is included in the diagram for the ease of reference. Data points can be obtained from the supplementary information [15,21,26,48,53,54,55,56,57,58,59,60] (Tables S5 and S6).

Subsequently, in order to understand the O_2_/N_2_ separation performance of CMSMs as reported in Table 2, quantification of both solubility and diffusivity of O_2_ and N_2_ was conducted. Based on the isotherm profile as illustrated in Figure 6a,b, at the specified pressure of O_2_ (0.21 bar) and N_2_ (0.79 bar), the amounts of O_2_ and N_2_ adsorbed in CMSMs are comparable. Nevertheless, 30 wt% PS-MFI in CMSMs demonstrated a sharp increase in both O_2_ and N_2_ adsorption. Due to a higher N_2_ adsorption in comparison to O_2_ adsorption (Figure 3a,b), it is generally challenging for the studied membranes to demonstrate an improved solubility selectivity as compared to diffusion selectivity. The selectivity in this context is defined based on ratio between O_2_ and N_2_. As indicated in Table S3, mixed-matrix CMSMs showed increased N_2_ solubility as compared to that of pure CMSM. The improvement in diffusion selectivity is plausibly caused by the smaller average micropore size in the mixed-matrix CMSMs (Table S3), which is capable of improving size-dependent discrimination of N_2_ and O_2_.

### 3.4. Performance Benchmarking with F_index_ and Robeson Upper Bound

The performance of mixed-matrix CMSMs in this study was benchmarked with the O_2_/N_2_ upper bound as described by Robeson. Based on the results, the performance of our membranes surpasses the 2008 upper bound limit for O_2_/N_2_ separation as shown in Figure 6. Thus, our mixed-matrix strategy proves effective in improving the gas separation performance substantially with reference to pure Matrimid^®^ CMSM. Although the performance of our CMSMs fall short to that of other pure CMSMs reported in the literature (Figure 6c), our strategy is much more straightforward as compared to the high-performance CMSMs developed using in-house synthesized polymers, which do not possess scalability potential. It is undeniable that the performance of our mixed-matrix CMSMs in this work is limited by the moderate decrease in O_2_/N_2_ selectivity. Nevertheless, the strong enhancement in O_2_ permeability reconciles this shortcoming. Furthermore, we studied the effectiveness of the zeolite nanocrystals using *F_index_*. *F_index_* is an empirical composite rating that was first initiated in 2018 to determine the particles’ effectiveness in mixed-matrix membrane [10]. Figure S4 and Table 2 summarize the calculated parameters. As a whole, PS-MFI at 20 and 30 wt% loading in CMSMs shows most promising performance for O_2_/N_2_ separation, with *F_index_* values of 2.86 and 3.35, respectively. As previously defined [10], these values place PS-MFI filler under the “competent” category for mixed-matrix CMSMs (Figure 6d and Table S6).

## 4. Conclusions

In this work, mixed-matrix CMSMs, incorporating various zeolite nanocrystals, were demonstrated using Matrimid^®^ 5218 as the polymer precursor. It can be observed that the addition of zeolite nanocrystals is feasible to elevate O_2_ permeability due to the presence of large micropore volumes on zeolite nanocrystals. Despite a slight decrease in O_2_/N_2_ selectivity, the O_2_/N_2_ Robeson upper bound can be successfully surpassed with the use of PS-MFI filler (at the loading of 20 and 30 wt%) in carbonized Matrimid^®^ 5218 membranes. Calculation of *F_index_* also demonstrates the feasibility of the studied mixed-matrix strategy to be competent for improving the O_2_/N_2_ separation performance. Future work that should be conducted is an investigation on the effect of zeolite nanocrystals on physical aging of CMSMs, which is an important consideration in practical applications. Besides, for application in industrial separation processes, the mixed-matrix CMSM should be fabricated into a thin-film composite membrane with an aid from porous supports to overcome its poor mechanical strength.

## Figures and Tables

**Table 1:** Surface area and pore volumes of SAPO-34, PS-MFI and SAPO-34 (determined using N_2_ physisorption (77 K)).

Sample	S_BET_ (m^2^ g^−1^) ^1^	S_LANGMUIR_ (m^2^ g^−1^) ^1^	S_MICRO_ (m^2^ g^−1^) ^2^	V_MICRO_ (cc g^−1^) ^2^	V_TOTAL_ (cc g^−1^) ^3^
SAPO-34	631	819	592	0.272	0.572
PS-MFI	493	667	397	0.195	0.563
ETS-10	359	518	319	0.163	0.358

^1^ Selection at P/P_o_ from 0.05 to 0.2 is required to calculate BET and Langmuir surface area (S_BET_ and S_LANGMUIR_). ^2^ Selection at P/P_o_ from 0.4 to 0.6 is required to calculate micropore surface area (S_MICRO_) and micropore volume (V_MICRO_). ^3^ Selection at *P/P_o_* = 0.99 is required to calculate the total pore volume (V_TOTAL_).

**Table 2:** O_2_/N_2_ permeation analysis of carbonized membrane. The 35 °C and 1 bar of air feed (O_2_/N_2_ = 21/79 vol/vol) was performed. *F_index_* calculated from Equation (3) was added for comparison.

CMSM	Thickness (μm) ^1^	O_2_ Permeability (barrer)	O_2_/N_2_ Selectivity	*F_index_*
Matrimid^®^ 5218	208 ± 28	5 ± 1	5.50 ± 0.10	-
10 wt% ETS-10	135 ± 13	79 ± 4	4.60 ± 0.56	1.74
20 wt% ETS-10	251 ± 20	126 ± 23	3.94 ± 0.49	1.33
30 wt% ETS-10	248 ± 18	141 ± 14	3.91 ± 0.14	1.40
10 wt% PS-MFI	138 ± 15	149 ± 1	4.86 ± 0.33	2.69
20 wt% PS-MFI	140 ± 28	199 ± 12	4.76 ± 0.03	2.86
30 wt% PS-MFI	157 ± 12	465 ± 5	4.46 ± 0.05	3.35
10 wt% SAPO-34	186 ± 38	119 ± 4	4.07 ± 0.10	1.46
20 wt% SAPO-34	184 ± 15	150 ± 2	4.46 ± 0.41	2.21
30 wt% SAPO-34	166 ± 2	176 ± 4	3.95 ± 0.22	1.68

^1^ The error bar on the thickness is reported as standard deviation.

## Data Availability

Not applicable.

## Supplementary Materials

The following are available online at https://www.mdpi.com/article/10.3390/membranes11070489/s1, Figure S1: O2 and N2 adsorption of ETS-10, PS-MFI and SAPO-34 at 25 °C; Figure S2: TGA analysis of ETS-10, PS-MFI and SAPO-34; Figure S3: Dubinin–Radushkevich (DR) plot for CMSMs and mixed-matrix CMSMs: (a) Matrimid, (b) 30 wt% ETS-10, (c) 30 wt% PS-MFI, and (d) 30 wt% SAPO-34; (e) CO2 adsorption of CMSMs and mixed-matrix CMSMs at 0 °C. The saturation pressure of CO2 (denoted as Po) is set at 26,141 torr; Figure S4: Performance of mixed-matrix CMSM with Findex indicated in the figure as reference. The Findex value can be obtained from Table 2, based on the calculation from Equation (5); Table S1: Fitting parameters for O2 and N2 for ETS-10, PS-MFI and SAPO-34 at 25 °C; Table S2: Fitting parameters for O2 and N2 for ETS-10, PS-MFI and SAPO-34 at 35 °C; Table S3: Porosity properties of CMSM and mixed-matrix CMSM; Table S4: Solubility and diffusivity of O2 and N2 of CMSM and mixed-matrix CMSM at 35 °C under the feed pressure of 1 bar (0.21 bar for O2 and 0.79 bar for N2); Table S5: Performance of pure CMSMs that have been reported in the literature for O2/N2 separation; Table S6: Performance of mixed-matrix CMSMs that have been reported in the literature for O_2_/N_2_ separation.
