# Supplementary material for: Enhanced Performance of Carbon Molecular Sieve Membranes Incorporating Zeolite Nanocrystals for Air Separation
Source: Membranes (Basel). 2021 Jun 29;11(7):489. doi: 10.3390/membranes11070489 (PMC8304111; doi:10.3390/membranes11070489)
Supplement: Supplementary file 1 [file membranes-11-00489-s001.zip › membranes-1280540-supplementary.pdf]

membranes

Supplementary Materials: Enhanced Performance of Carbon Molecular Sieve Membranes Incorporating Zeolite Nanocrystals for Air Separation

Chong Yang Chuah [1], Kunli Goh [1] and Tae-Hyun Bae [2,*]

[1] Singapore Membrane Technology Centre, Nanyang Environment and Water Research Institute, Nanyang Technological University, Singapore 637141 Singapore; chongyang.chuah@ntu.edu.sg (C.Y.C.)
[2] Department of Chemical and Biomolecular Engineering (BK21 four), Korea Advanced Institute of Science and Technology, Daejeon 34141, Korea
* Correspondence: thbae@kaist.ac.kr (T-H.B.)

SUMMARY

Number of pages: 8

Number of Figures: 4

Number of Tables: 6

Table S1. Fitting parameters for O_2 and N_2 adsorptions for ETS-10, PS-MFI and SAPO-34 at 25 °C.

Sample	*gas*	q_{sat}	*b*	R^2 value
**ETS-10**	O_2	2.272	0.08588	1
	N_2	2.272	0.2268	0.9961
**PS-MFI**	O_2	3.774	0.04305	1
	N_2	3.774	0.04603	0.9999
**SAPO-34**	O_2	5.470	0.03192	0.9967
	N_2	5.470	0.03722	0.9966

Table S2. Fitting parameters for O_2 and N_2 adsorptions for ETS-10, PS-MFI and SAPO-34 at 35 °C.

Sample	*gas*	q_{sat}	*b*	R^2 value
**ETS-10**	O_2	2.241	0.07729	1
	N_2	2.241	0.1936	0.9991
**PS-MFI**	O_2	3.845	0.03399	0.9995
	N_2	3.845	0.03631	0.9999
**SAPO-34**	O_2	5.333	0.02703	0.9979
	N_2	5.333	0.03108	0.9978

Table S3. Porosity properties of CMSM and mixed-matrix CMSMs.

Membrane	Average micropore size (nm)	Micropore volume (cc)	Micropore surface area (m^2/g)
**Matrimid® 5218**	0.897	0.191	519
**30 wt% ETS-10**	0.547	0.209	556
**30 wt% PS-MFI**	0.673	0.241	643
**30 wt% SAPO-34**	0.701	0.260	692

Table S4. Solubility and diffusivity of O_2 and N_2 of CMSM and mixed-matrix CMSMs at 35 ^oC under the feed pressure of 1 bar (0.21 bar for O_2 and 0.79 bar for N_2).

Membrane	Density (g/cm^3)	O_2 solubility (mol/m^3-bar)	N_2 solubility (mol/m^3-bar)	O_2 diffusivity (m^2/s)	N_2 diffusivity (m^2/s)
**Matrimid® 5218**	1.288	143	86	2.65×10^{-13}	8.00×10^{-14}
**30 wt% ETS-10**	1.491	152	88	7.07×10^{-12}	3.11×10^{-12}
**30 wt% PS-MFI**	1.439	200	156	1.76×10^{-11}	5.08×10^{-12}
**30 wt% SAPO-34**	1.478	137	117	9.76×10^{-12}	2.88×10^{-12}

Table S5. Performance of pure CMSMs that have been reported in the literature for O_2/N_2 separation.

Membrane	Density (g/cm^3)	O_2 solubility (mol/m^3-bar)	N_2 solubility (mol/m^3-bar)	O_2 diffusivity (m^2/s)	N_2 diffusivity (m^2/s)
**BTPA-ODA**	550 ^{o}C, Argon	25 ^{o}C, pure gas	500	10.4	[1]
**Kapton**	550 ^{o}C, vacuum	25 ^{o}C, pure gas	45	4.4	[2]
**Kapton-1**	600 ^{o}C, vacuum	35 ^{o}C, pure gas	383	4.7	[3]
**Matrimid**	550 ^{o}C, Argon	35 ^{o}C, mixed gas (21/79)	5	5.5	This work
**Matrimid-1**	475 ^{o}C, vacuum	25 ^{o}C, pure gas	4	4.5	[2]
**Matrimid-2**	550 ^{o}C, vacuum	35 ^{o}C, 10 atm, pure gas	280	4.8	[4]
**Br-Matrimid-2**	550 ^{o}C, vacuum	35 ^{o}C, 10 atm, pure gas	850	5.1	[4]
**ODPA-ODA**	650 ^{o}C	50 ^{o}C, pure gas	70.3	6.0	[5]
**ODPA-ODA-1**	650 ^{o}C	50 ^{o}C, mixed gas (21:79)	33.2	5.1	[5]
**P84**	600 ^{o}C, Nitrogen	Pure gas	72	9.3	[6]
**PEI**	550 ^{o}C, vacuum	25 ^{o}C, pure gas	19	2.1	[7]
**PEI-1**	500 ^{o}C, vacuum	26 ^{o}C, pure gas	12	3.9	[8]
**Polypyrrole**	500 ^{o}C, Nitrogen	35 ^{o}C, pure gas	815	8.2	[9]
**Poly(amino imide)**	150 ^{o}C, Nitrogen	35 ^{o}C, pure gas	1	6.1	[9]
**PPO-2**	650 ^{o}C, vacuum	25 ^{o}C, pure gas	55	11.4	[10]
**TMSPPO80**	650 ^{o}C, vacuum	25 ^{o}C, pure gas	125	10	[10]

Note: BTDA: Benzophenone tetracarboxylic dianhydride; ODA = 4,4′-oxydianiline; PPO = poly(p-phenylene oxide); TMS = chlorotrimethylsilane

Table S6. Performance of mixed-matrix CMSMs that have been reported in the literature for O_2/N_2 separation.

CMSM	Loading (wt%)	Measurement condition	O_2 permeability (barrer)	O_2/N_2 selectivity	F_{index}	Ref.
**ETS-10 (Matrimid)**	30	35 °C, mixed gas (21/79)	141	3.9	1.40	This work
**PS-MFI (Matrimid)**	30	35 °C, mixed gas (21/79)	465	4.5	3.35	This work
**SAPO-34 (Matrimid)**	30	35 °C, mixed gas (21/79)	170	4.0	1.68	This work
**zeolite 5A-100 nm (Matrimid)**	10	35 °C, mixed gas (21/79)	77	4.0	0.83	[11]
**zeolite 5A-100 nm (Matrimid)**	20	35 °C, mixed gas (21/79)	168	3.7	1.17	[11]
**zeolite 5A-1 μm (Matrimid)**	10	35 °C, mixed gas (21/79)	98	3.9	1.35	[11]
**zeolite 5A-1 μm (Matrimid)**	20	35 °C, mixed gas (21/79)	121	3.9	1.14	[11]
**zeolite 5A (Matrimid)**	10	35 °C, mixed gas (21/79)	84	4.4	1.45	[12]
**zeolite 5A (Matrimid)**	20	35 °C, mixed gas (21/79)	142	4.2	1.73	[12]
**H-zeolite 5A (Matrimid)**	10	35 °C, mixed gas (21/79)	151	4.4	2.03	[12]
**H-zeolite 5A (Matrimid)**	20	35 °C, mixed gas (21/79)	185	4.0	1.64	[12]
**ZSM-5 (ODPA-ODA)**	0.5	50 °C, pure gas	13	4.2	-3.74	[5]
**ZSM-5 (ODPA-ODA)**	0.5	50 °C, mixed gas (21/79)	4.2	4.1	-5.00	[5]

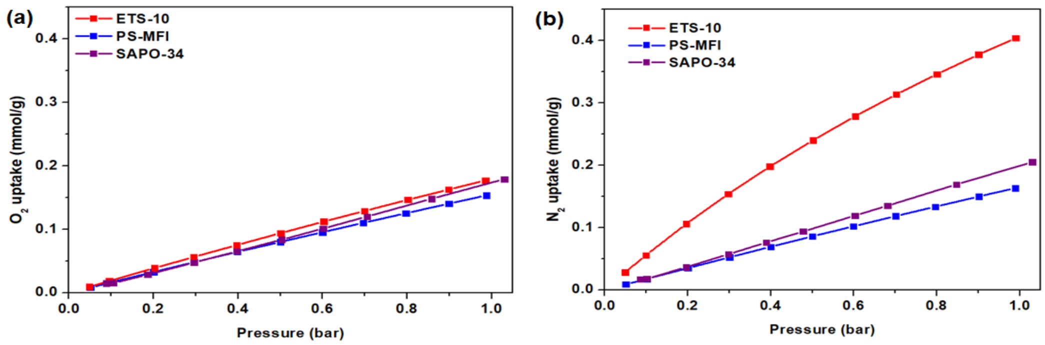

Figure S1. (a, b) O_2 and N_2 adsorption of ETS-10, PS-MFI and SAPO-34 fillers at 25 °C

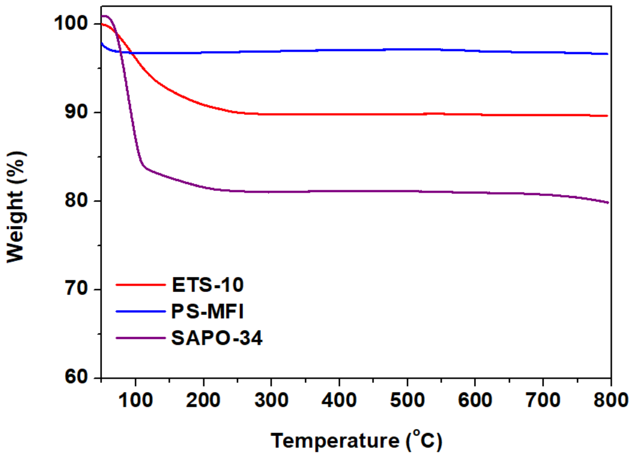

Figure S2. TGA analysis of ETS-10, PS-MFI and SAPO-34 fillers.

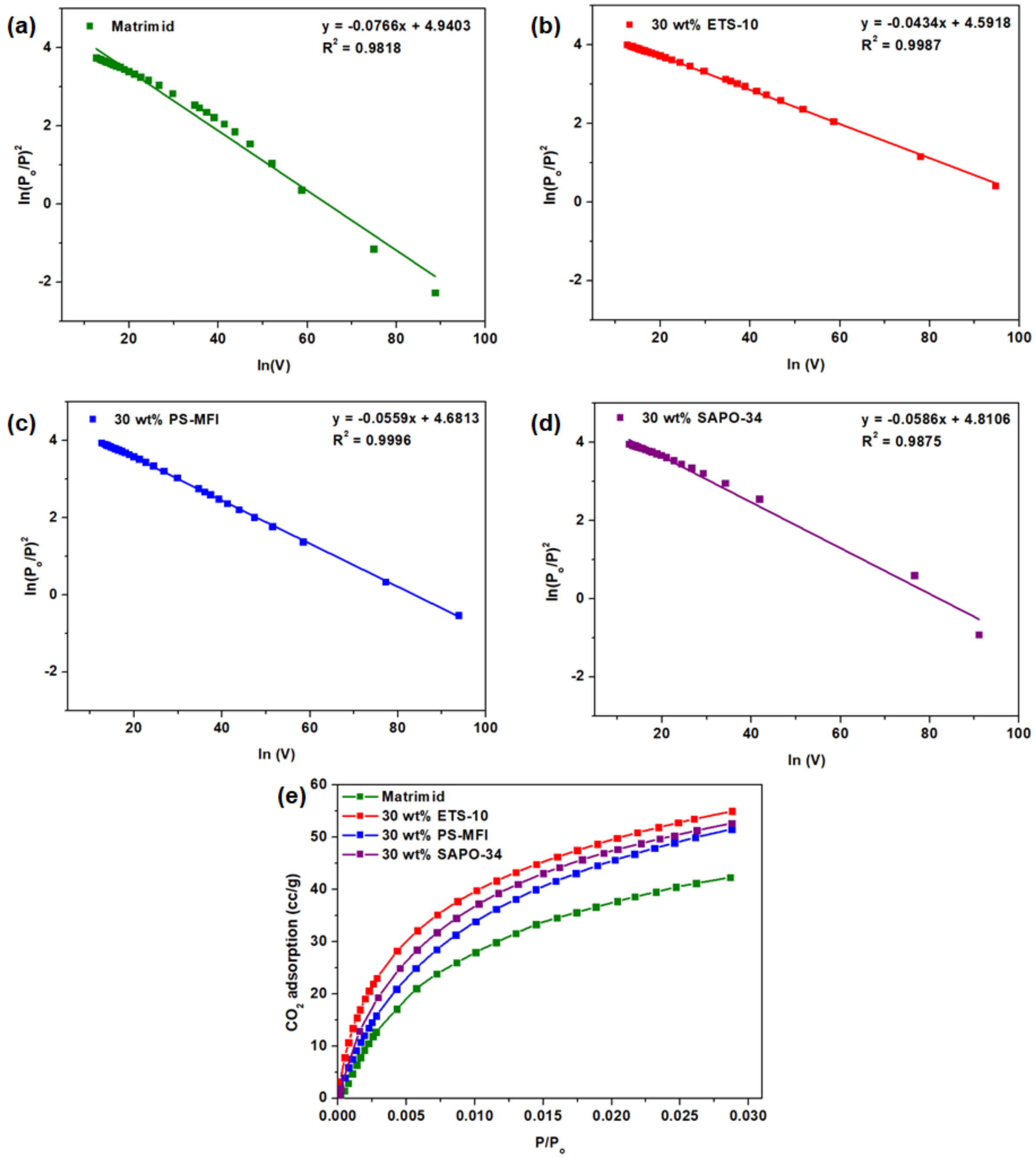

Figure S3 Dubinin-Radushkevich (DR) plot for CMSMs and mixed-matrix CMSMs: **(a)** Matrimid, **(b)** 30 wt% ETS-10, **(c)** 30 wt% PS-MFI, and **(d)** 30 wt% SAPO-34; **(e)** CO_2 adsorption of CMSMs and mixed-matrix CMSMs at 0 °C. The saturation pressure of CO_2 (denoted as P_o) is set at 26,141 torr

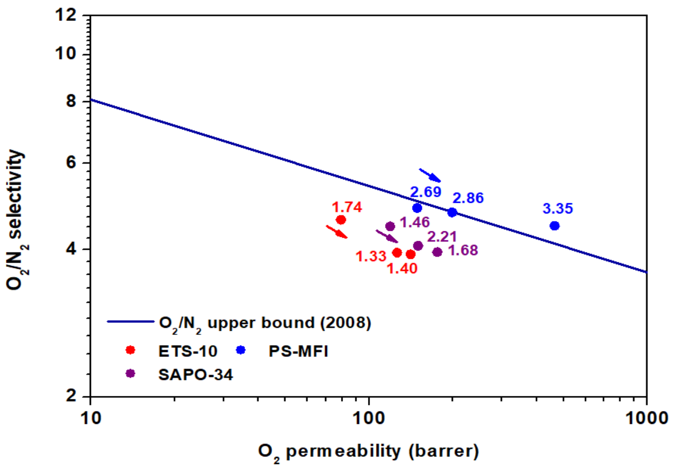

Figure S4 Performance of mixed-matrix CMSMs with F_{index} values indicated. The F_{index} values can be obtained from **Table 2**, based on calculations from equation (5)

References

1. Y.K. Kim, H.B. Park, Y.M. Lee, Carbon molecular sieve membranes derived from thermally labile polymer containing blend polymers and their gas separation properties, J. Membr. Sci., 243 (2004) 9-17.
2. A. Fuertes, D. Nevskaia, T. Centeno, Carbon composite membranes from Matrimid® and Kapton® polyimides for gas separation, Micropor. Mesopor. Mater., 33 (1999) 115-125.
3. H. Suda, K. Haraya, Gas permeation through micropores of carbon molecular sieve membranes derived from Kapton polyimide, J. Phys. Chem. B, 101 (1997) 3988-3994.
4. Y. Xiao, Y. Dai, T.-S. Chung, M.D. Guiver, Effects of brominating Matrimid polyimide on the physical and gas transport properties of derived carbon membranes, Macromolecules, 38 (2005) 10042-10049.
5. B. Zhang, Y. Wu, Y. Lu, T. Wang, X. Jian, J. Qiu, Preparation and characterization of carbon and carbon/zeolite membranes from ODPA–ODA type polyetherimide, J. Membr. Sci., 474 (2015) 114-121.
6. J. Barsema, J. Balster, V. Jordan, N. Van der Vegt, M. Wessling, Functionalized carbon molecular sieve membranes containing Ag-nanoclusters, J. Membr. Sci., 219 (2003) 47-57.
7. A.K. Itta, H.-H. Tseng, M.-Y. Wey, Effect of dry/wet-phase inversion method on fabricating polyetherimide-derived CMS membrane for H_2/N_2 separation, Int. J. Hydrog. Energy, 35 (2010) 1650-1658.
8. P.S. Rao, M.-Y. Wey, H.-H. Tseng, I.A. Kumar, T.-H. Weng, A comparison of carbon/nanotube molecular sieve membranes with polymer blend carbon molecular sieve membranes for the gas permeation application, Micropor. Mesopor. Mater., 113 (2008) 499-510.
9. H. Kita, M. Yoshino, K. Tanaka, K.-i. Okamoto, Gas permselectivity of carbonized polypyrrolone membrane, Chem. Commun., (1997) 1051-1052.
10. M. Yoshimune, I. Fujiwara, K. Haraya, Carbon molecular sieve membranes derived from trimethylsilyl substituted poly(phenylene oxide) for gas separation, Carbon, 45 (2007) 553-560.
11. W. Li, C.Y. Chuah, S. Kwon, K. Goh, R. Wang, K. Na, T.-H. Bae, Nanosizing zeolite 5A fillers in mixed-matrix carbon molecular sieve membranes to improve gas separation performance, Chem. Eng. J. Adv., 2 (2020) 100016.
12. W. Li, C.Y. Chuah, T.-H. Bae, Hierarchical 5A Zeolite-Containing Carbon Molecular Sieve Membranes for O_2/N_2 Separation, Membr. J., 30 (2020) 260-268.
